# Generalized Nonlinear Chirp Scaling Algorithm for High-Resolution Highly Squint SAR Imaging

**DOI:** 10.3390/s17112568

**Published:** 2017-11-07

**Authors:** Tianzhu Yi, Zhihua He, Feng He, Zhen Dong, Manqing Wu

**Affiliations:** 1 School of Electronic Science and Engineering, National University of Defense Technology, Sanyi Avenue, Changsha 410073, China; skynismile@163.com (Z.H.); hefeng@nudt.edu.cn (F.H.); dongzhen@nudt.edu.cn (Z.D.); 2 China Electronics Technology Group Corporation (CETC), China Academy of Electronics and Information Technology, Beijing 100846, China; wmq_cetc@163.com

**Keywords:** synthetic aperture radar (SAR), linear range walk correction (LRWC), generalized nonlinear chirp scaling (GNLCS), azimuth depth of focusing (ADOF), focusing precision

## Abstract

This paper presents a modified approach for high-resolution, highly squint synthetic aperture radar (SAR) data processing. Several nonlinear chirp scaling (NLCS) algorithms have been proposed to solve the azimuth variance of the frequency modulation rates that are caused by the linear range walk correction (LRWC). However, the azimuth depth of focusing (ADOF) is not handled well by these algorithms. The generalized nonlinear chirp scaling (GNLCS) algorithm that is proposed in this paper uses the method of series reverse (MSR) to improve the ADOF and focusing precision. It also introduces a high order processing kernel to avoid the range block processing. Simulation results show that the GNLCS algorithm can enlarge the ADOF and focusing precision for high-resolution highly squint SAR data.

## 1. Introduction

Synthetic aperture radar (SAR) has been widely used as a remote sensing tool as it can provide the high-resolution images of the interested area during a mission, nearly regardless of weather and time. Side-looking SAR, with the restriction of the antenna beam pointing direction, can only be applied in some special areas [1]. The squint SAR, which has an offset angle between the antenna beam pointing direction and flight path, can be flexibly applied in forehead observation [2,3,4,5,6], for example. The squint SAR can enhance the survival probability since it accomplishes the monitoring mission without going through the battlefield. It also has the advantages of associating with multi-mode to achieve some special applications, such as video SAR [7,8,9,10,11,12] and increasing revisiting times of the interested area [11]. When compared to the perpendicular broadside SAR, the squint SAR is more attractive and widely used in the monostatic system, because it provides more flexibility for observing missions of SAR [13].

A number of outstanding and efficient frequency-domain algorithms have been proposed to solve the perpendicular broadside stripmap SAR imaging in the last fifty years, such as range Doppler algorithm (RDA), ω−k algorithm, chirp scaling algorithm (CSA), and frequency scaling algorithm (FSA) [14]. Both the RDA and ω−k have a low efficiency because of the interpolation process to accomplish the range cell migration correction (RCMC) [15,16]. By using the chirp scaling function, CSA and FSA reach a relative faster imaging process compared to RDA and ω−k. Since the RDA, CSA, and FSA are built and derived under the zero Doppler assumption, they can only process the echo data with a squint angle varying from 0° to 5°. The focusing performances of the aforementioned three conventional imaging algorithms decrease with the increasing of the squint angle. In comparison with the perpendicular broadside SAR, new complexities of signal properties are introduced in the squint SAR [1]. The squint SAR geometry is also different from the conventional broadside one, which results in the difficulties in focusing precisely. The key problems of processing squint SAR data are the compensations of two-dimensional (2D) spatial-variant range cell migration (RCM), and azimuth-variance of Doppler coefficients [17].

A number of imaging algorithms have been presented for the squint SAR data. Back projection (BP) algorithm is the most precise imaging algorithm when assuming that the trajectory is obtained accurately. However, it has the heaviest computational burden among the imaging algorithms [18]. Extended ω−k algorithm can achieve the highest accuracy among all of the frequency domain processing algorithms. However, its computational burden is much heavier than that of the nonlinear CS algorithm (NLCSA) [19]. Although the NLCSA is capable of processing the squint SAR data, it is only available to low/medium squint angle SAR [20]. Then, the extended NLCSA (ENLCSA), which introduces a new perturbation function to equalize the frequency modulation (FM) rates [1], is proposed to deal with the highly squint data whose squint angle can be up to 65°. However, the azimuth depth of focus (ADOF) for the ENLCSA decreases when the distance of targets deviating from the reference azimuth position increases. With a 60° squint angle and 1m resolution, the ADOF can only reach 200 m by NLCSA. It is obvious that the ENLCSA cannot be used in the SAR mission with high resolution and high squint angle requirements. In this paper, a new method is proposed to solve the deteriorations caused by azimuth variant FM rates for high-resolution and highly squint SAR. Furthermore, modification is also carried out in range direction to avoid the range sub-block process with wide swath data.

The remaining part of the paper is organized as follows. Section 2 gives the model of the signal acquisition for squint SAR and illustrates the processing method of generalized nonlinear chirp scaling algorithm (GNLCSA); In Section 3, the dot-matrix simulation under high-resolution highly squint SAR configuration is carried out to validate the proposed algorithm; and, a summary is performed in Section 4.

## 2. GNLCSA

### 2.1. Preprocess in Range Direction by Generalized Chirp Scaling

**Figure 1:**
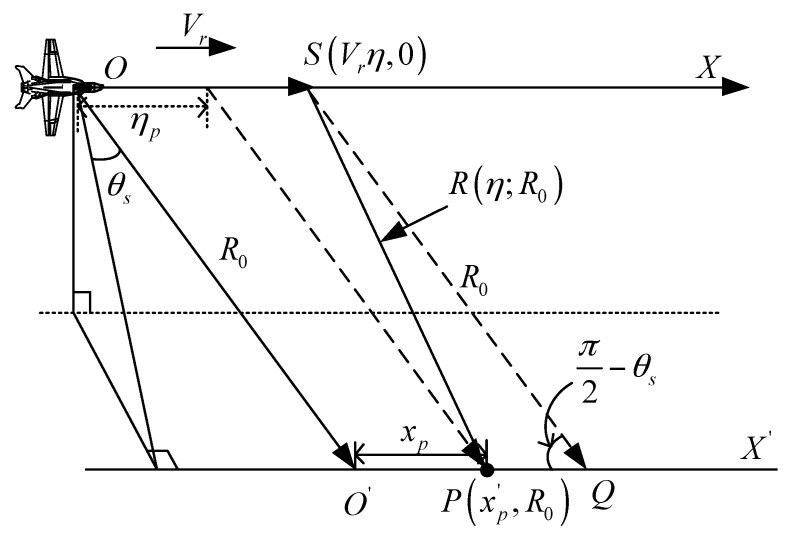
Geometry of squint synthetic aperture radar (SAR). The geometry of a general squint SAR provides the model of the signal acquisition. The radar flies along the X-axis with the velocity of $V_{r}$. $\vec{OO^{\prime }}$ is the vector of the beam illumination. The angle of which away from the X-axis is ($\frac{\pi }{2}-\theta_{s}$), where $\theta_{s}$ is the squint angle. $R_{0}$ is the slant range between the sensor and the boresight intersection.

Assume that target P is an object of the observation. In Figure 1, $R\left(\eta ;R_{0}\right)$ is the range between target P and the sensor at the azimuth time of η. $x_{p}$ is defined as the separation distance bias between the footprint of the beam center and target P.

In △SQP, based on the cosine theorem, the instantaneous slant range $R\left(\eta ;R_{0}\right)$ can be given by the following equation:

(1) $$R\left(\eta ;R_{0}\right)=\sqrt{R_{0}^{2}+{\left(V_{r}\eta -x_{p}\right)}^{2}+2R_{0}\left(V_{r}\eta -x_{p}\right)\cos\left(\frac{\pi }{2}-\theta_{s}\right)}$$

Assume that a linear FM (LFM) pulse is transmitted by the sensor, and the signal reflected from target P can be expressed as:

(2) $$\begin{array}{cc} s_{r}\left(\tau ,\eta \right) & =\omega_{r}\left(\tau -2R\left(\eta ;R_{0}\right)/c\right)\omega_{a}\left(\eta -\eta_{p}\right)\cdot \exp\left[-j\frac{4\pi }{\lambda }R\left(\eta ;R_{0}\right)\right]\\ & \cdot \exp\left[-j\pi K_{r}{\left(\tau -2R\left(\eta ;R_{0}\right)/c\right)}^{2}\right] \end{array}$$

where τ is the fast time; $\eta_{p}$ is the center Doppler moment of target P. c is the speed of light; $\omega_{r}\left(•\right)$ and $\omega_{a}\left(•\right)$ are the antenna patterns in range and azimuth directions, respectively [18]. $f_{0}$ is the carrier frequency of the radar. $\lambda =f_{0}/c$ represents the wavelength of the carrier frequency. $K_{r}$ is the chirp rate of the LFM signal. By Taylor expansion, $R\left(\eta ;R_{0}\right)$ can be further approximated as:

(3) $$\begin{array}{cc} R\left(\eta ;R_{0}\right) & \approx R_{0}+\frac{\cos^{2}\theta_{s}{\left(V_{r}\eta -x_{p}\right)}^{2}}{2R_{0}}+\frac{\sin\theta_{s}\cos^{2}\theta_{s}}{2R_{0}^{2}}{\left(V_{r}\eta -x_{p}\right)}^{3}-\frac{\cos^{2}\theta_{s}\left(-3+5\cos\left(2\theta_{s}\right)\right){\left(V_{r}\eta -x_{p}\right)}^{4}}{16R_{0}^{3}}\\ & -\frac{\cos^{2}\theta_{s}\left(-1+7\cos\left(2\theta_{s}\right)\sin\theta_{s}\right){\left(V_{r}\eta -x_{p}\right)}^{3}}{16R_{0}^{4}}-\left(V_{r}\eta -x_{p}\right)\sin\theta_{s}+O{\left[\eta \right]}^{5} \end{array}$$

In Equation (3), the term $V_{r}\eta \sin\theta_{s}$ represents linear range walk, which can be compensated by the function of $H_{1}$:

(4) $$H_{1}\left(f_{\tau },\eta \right)=\exp\left(-j4\pi \frac{f_{\tau }+f_{0}}{c}V_{r}\eta \sin\theta_{s}\right)$$

where $f_{\tau }$ is the range frequency. After the linear range walk correction (LRWC) in range frequency domain, the signal can be given as:

(5) $$\begin{array}{cc} s_{1}\left(f_{\tau },\eta \right) & =W_{r}\left(f_{\tau }\right)\omega_{a}\left(\eta -\eta_{p}\right)\exp\left(-j4\pi \frac{f_{\tau }+f_{0}}{c}V_{r}\eta_{p}\sin\theta_{s}\right)\exp\left(-j\pi \frac{f_{\tau }^{2}}{K_{r}}\right)\\ & \cdot \exp\left(-j4\pi \frac{f_{\tau }+f_{0}}{c}\sqrt{R_{0}^{2}+\cos^{2}\theta_{s}{\left(V_{r}\eta -x_{p}\right)}^{2}}\right)\\ & \cdot \exp\left(-j4\pi \frac{f_{\tau }+f_{0}}{c}\frac{\sin\theta_{s}\cos^{2}\theta_{s}}{2R_{0}^{2}}{\left(V_{r}\eta -x_{p}\right)}^{3}\right) \end{array}$$

**Figure 2:**
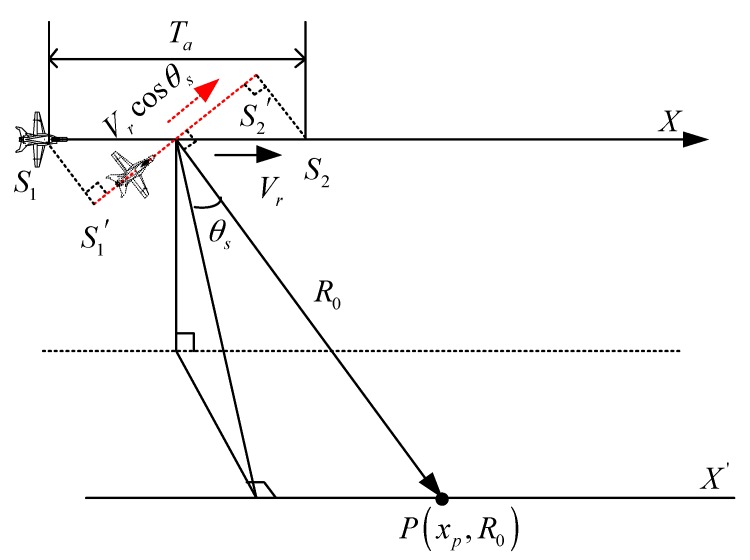
Geometry of squint SAR after the linear range walk correction (LRWC). The red dot line represents the equivalent trajectory of the platform after the LRWC.

Another way to understand LRWC is shown in the Figure 2. $T_{a}$ is the synthetic aperture time of radar in the original sampling geometry. The flight path during the synthetic aperture time of target P is $\vec{S_{1}S_{2}}$. After the LRWC, its equivalent trajectory turns to $\vec{S_{1}{}^{\prime }S_{2}{}^{\prime }}$. Accordingly, the equivalent velocity becomes $V_{r}\cos\theta_{s}$. After the LRWC, the signal model can be treated as the “broadside SAR”. The difference between the broadside SAR and the squint SAR after the LRWC is that the nearest slant range of the latter also turns to $\left(R_{0}+V_{r}\eta_{p}\sin\theta_{s}\right)$, which means that the Doppler rates of the squint SAR after the LRWC are azimuth variant. We define that:

(6) $$R_{LRWC}=R_{0}+V_{r}\eta_{p}\sin\theta_{s}$$

With an inverse fast Fourier transform (IFFT) along range direction, we reach the signal of Equation (5) in 2D time domain:

(7) $$\begin{array}{cc} s_{1}^{\prime }\left(\tau ,\eta \right) & =\omega_{r}\left(\tau -2\left(R\left(\eta ;R_{0}\right)+V_{r}\eta \sin\theta_{s}\right)/c\right)\omega_{a}\left(\eta -\eta_{p}\right)\\ & \cdot \exp\left[-j4\pi \frac{f_{0}}{c}\left(R\left(\eta ;R_{0}\right)+V_{r}\eta \sin\theta_{s}\right)\right]\exp\left(-j\pi K_{r}{\left(\tau -2\left(R\left(\eta ;R_{0}\right)+V_{r}\eta \sin\theta_{s}\right)/c\right)}^{2}\right) \end{array}$$

We can obtain the range history of the target $\left(R\left(\eta ;R_{0}\right)+V_{r}\eta \sin\theta_{s}\right)$ after the LRWC, which is different from that of the raw squint data. As shown in Figure 2, the LRWC for squint data brings more benefits when processes the range cell migration (RCMC), range compression (RC), and the secondary range compression (SRC). In general, to reach the requirement of high-resolution in range direction, the pulse with wide bandwidth is needed in the SAR system. References [18,21] explain in detail that the conventional CSA and RDA are not capable of processing the RCMC/RC/SRC with wide bandwidth. Due to the limited space, this paper will not repeat the methods of precise focusing along the range direction for the wide bandwidth SAR. 

### 2.2. Azimuth Filter and Coefficients

A 2D fast Fourier transform (FFT) yields the signal of Equation (7) in the 2D frequency domain:

(8) $$s_{1}\left(f_{\tau },f_{\eta }\right)=W_{r}\left(f_{\tau }\right)W_{a}\left(f_{\eta }\right)\cdot \exp\left(-j2\pi f_{\eta }\eta_{p}\right)\cdot \exp\left(-j4\pi \frac{\left(f_{\tau }+f_{0}\right)}{c}x_{p}\sin\theta_{s}\right)\cdot \exp\left(j\Phi \left(f_{\tau },f_{\eta }\right)\right)$$

The first exponential term implies the position of target, and the second one indicates the offset of the target’s position, which can be corrected after the accomplishment of azimuth compression. $\Phi \left(f_{\tau },f_{\eta }\right)$ represents the phase coupling between the range and azimuth directions.

(9) $$\Phi \left(f_{r},f_{\eta }\right)\approx \phi_{0}\left(f_{\eta }\right)R_{0}+\sum_{i=1}^{n}\phi_{i}\left(f_{\eta },R_{0}\right)f_{\tau }^{i}$$

In Section 2, it has been discussed that the processing of range dimension is accessible in the References [18,21]. It is considered that $\sum_{i=1}^{n}\phi_{i}\left(f_{\eta },R_{0}\right)f_{\tau }^{i}$ is eliminated after the processing of range dimension, so the coefficients $\phi_{i}\left(i=1,2,\cdots ,n\right)$ are not discussed again in this paper. The $\phi_{0}\left(f_{\eta }\right)$ is shown as the following equation:

(10) $$\begin{array}{cc} \phi_{0}\left(f_{\eta }\right) & \approx -\frac{4\pi }{\lambda }-\frac{2\pi \eta_{p}}{R_{0}}f_{\eta }+\frac{\pi }{\lambda }\frac{\sec^{2}\theta_{s}}{2V_{r}^{2}}f_{\eta }^{2}+\pi \frac{\sin\theta_{s}}{\cos^{4}\theta_{s}}\frac{\lambda^{2}}{4V_{r}^{3}}f_{\eta }^{3}-\pi \lambda^{3}\frac{\left(-3+2\cos\left(2\theta_{s}\right)\right)}{32V_{r}^{4}\cos^{6}\theta_{s}}f_{\eta }^{4}\\ & -\pi \lambda^{4}\frac{\left(-6\sin\theta_{s}+\sin\left(3\theta_{s}\right)\right)}{64V_{r}^{5}\cos^{8}\theta_{s}}f_{\eta }^{5}+O{\left[f_{\eta }\right]}^{5}\\ & =\sum_{i=0}^{5}\varphi_{i}f_{\eta }^{i}+O{\left[f_{\eta }\right]}^{5} \end{array}$$

After the RCMC/SRC/RC processing of the signal in Equation (8), the signal in RD domain is expressed as:

(11) $$s_{2}\left(\tau ,f_{\eta }\right)=\omega_{r}\left(\tau \right)W_{a}\left(f_{\eta }\right)\cdot \mathrm{sinc}\left(\tau -2R_{LRWC}/c\right)\cdot \exp\left(j\theta_{2}\left(f_{\eta }\right)\right)$$

where $\theta_{2}\left(f_{\eta }\right)$ is $\phi_{0}\left(f_{\eta }\right)R_{0}$. The coefficients $\varphi_{0}∼\varphi_{5}$ are as follows:

(12) $$\{\begin{array}{cc} \varphi_{0}=-\frac{4\pi }{\lambda }; & \varphi_{1}=-\frac{2\pi \eta_{p}}{R_{0}}\\ \varphi_{2}=\pi \lambda \frac{\sec^{2}\theta_{s}}{2V_{r}^{2}}; & \varphi_{3}=\pi \lambda^{2}\frac{\sin\theta_{s}}{\cos^{4}\theta_{s}}\frac{1}{4V_{r}^{3}}\\ \varphi_{4}=-\pi \lambda^{3}\frac{\left(-3+2\cos\left(2\theta_{s}\right)\right)}{32V_{r}^{4}\cos^{6}\theta_{s}}; & \varphi_{5}=-\pi \lambda^{4}\frac{\left(-6\sin\theta_{s}+\sin\left(3\theta_{s}\right)\right)}{64V_{r}^{5}\cos^{8}\theta_{s}} \end{array}$$

Also, we define the coefficients $\varphi_{i,LRWC}$ and $\varphi_{i,s}\left(i=3,4,5\cdots \right)$, respectively.

(13) $$\left\{ \begin{array}{l} \varphi_{i,LRWC}\left(R_{LRWC}\right)=\varphi_{i}R_{LRWC}\\ \varphi_{i,s}=-\varphi_{i}V_{r}\sin\theta_{s} \end{array}\right.$$

Substituting the Equation (6) to $\phi_{0}\left(f_{\eta }\right)R_{0}$, we can rewrite $\theta_{2}\left(f_{\eta }\right)$:

(14) $$\theta_{2}\left(f_{\eta }\right)=-\frac{4\pi \left(R_{LRWC}-V_{r}\eta_{p}\sin\theta_{s}\right)}{\lambda }-2\pi \eta_{p}f_{\eta }+\varphi_{2}R_{0}f_{\eta }^{2}+\sum_{i=3}^{n}\left(\varphi_{i,LRWC}+\varphi_{i,s}\eta_{p}\right)f_{\eta }^{i}$$

In this paper, the method of series reversion (MSR) is used to enhance the focusing precision of the algorithm and the ADOF for the high-resolution highly squint SAR. The first step of azimuth processing is the compensation of high order phase. The effect of high order phase filter (HOPF) is consistent with the one in [4], which is given as follows:

(15) $$H_{2}\left(f_{\eta }\right)=\exp\left[-j\sum_{i=3}^{n}\varphi_{i,LRWC}\left(R_{0}\right)f_{\eta }^{i}\right]$$

Filtered by $H_{2}\left(f_{\eta }\right)$, the signal of Equation (8) can be rewritten as:

(16) $$s_{3}\left(\tau ,f_{\eta }\right)\approx \omega_{r}\left(\tau \right)W_{a}\left(f_{\eta }\right)\cdot \mathrm{sinc}\left(\tau -2R_{LRWC}/c\right)\cdot \exp\left(j\left(-2\pi \eta_{p}f_{\eta }+\varphi_{2}R_{0}f_{\eta }^{2}+\sum_{i=3}^{n}\varphi_{i,s}\eta_{p}f_{\eta }^{i}\right)\right)$$

$\varphi_{2}R_{0}$ can be rewritten as $\varphi_{2}R_{0}=-\frac{\pi }{-2V_{r}^{2}\cos^{2}\theta_{s}/\lambda /R_{0}}=-\frac{\pi }{K_{a}}$, where $K_{a}$ is the FM rates along azimuth direction. In Section 2, we have discussed that the equivalent nearest slant range is $R_{LRWC}$ after the LRWC. Substitute the Equation (6) to the expression of $K_{a}$ and expand $K_{a}$ near $\eta_{p}=0$:

(17) $$\begin{array}{cc} K_{a} & =-\frac{2V_{r}^{2}\cos^{2}\theta_{s}}{\lambda \left(R_{LRWC}-V_{r}\eta_{p}\sin\theta_{s}\right)}\\ & \approx -\frac{2V_{r}^{2}\cos^{2}\theta_{s}}{\lambda R_{LRWC}}-\frac{2V_{r}^{2}\cos^{2}\theta_{s}}{\lambda R_{LRWC}}\frac{V_{r}\sin\theta_{s}}{R_{LRWC}}\eta_{p}-\frac{2V_{r}^{2}\cos^{2}\theta_{s}}{\lambda R_{LRWC}}\frac{V_{r}^{2}\sin^{2}\theta_{s}}{R_{LRWC}^{2}}\eta_{p}^{2}\\ & -\frac{2V_{r}^{2}\cos^{2}\theta_{s}}{\lambda R_{LRWC}}\frac{V_{r}^{3}\sin^{3}\theta_{s}}{R_{LRWC}^{3}}\eta_{p}^{3}+O{\left[\eta_{p}\right]}^{4}\\ & =K_{LRWC}\left(1+K_{s}\eta_{p}+K_{s}^{2}\eta_{p}^{2}+K_{s}^{3}\eta_{p}^{3}\right)+O{\left[\eta_{p}\right]}^{4} \end{array}$$

(18) $$\begin{array}{l} K_{LRWC}=-\frac{2V_{r}^{2}\cos^{2}\theta_{s}}{\lambda R_{LRWC}}\\ K_{s}=\frac{V_{r}\sin\theta_{s}}{R_{LRWC}} \end{array}$$

It turns that the FM rates of different slant range along the azimuth direction are azimuth variant according to the Equations (16)–(18). This paper adds a degree of freedom to achieve a coincident FM rate form of different slant range along the azimuth direction. The turbulent function is expressed as:

(19) $$H_{3}\left(f_{\eta }\right)=\exp\left(j\pi \sum_{i=3}^{n}X_{i}f_{\eta }^{i}\right)$$

Assume the coefficients $\varphi_{i,s}^{\prime }\left(i=3,4,5\right)$ are proportional to $\varphi_{i,s}$ as follows:

(20) $$\varphi_{i,s}^{\prime }=\varphi_{i,s}/\pi$$

Multiplying (19) with (16) results in:

(21) $$s_{4}\left(\tau ,f_{\eta }\right)\approx \omega_{r}\left(\tau \right)W_{a}\left(f_{\eta }\right)\cdot \mathrm{sinc}\left(\tau -2R_{LRWC}/c\right)\cdot \exp\left(j\left(-2\pi \eta_{p}f_{\eta }-\frac{\pi }{K_{a}}f_{\eta }^{2}+\sum_{i=3}^{n}\left(\varphi_{i,s}^{\prime }\eta_{p}+X_{i}\right)f_{\eta }^{i}\right)\right)$$

The MSR is applied to calculate the solution of stationary phase (SP) of the Equation (21). To ensure the precision of the solution, this paper reserves a second order form for the solution, which is given as:

(22) $$f_{\eta ,SP}=K_{a}\left(\eta -\eta_{p}\right)+\frac{3}{2}k_{3}K_{a}^{3}{\left(\eta -\eta_{p}\right)}^{2}$$

We can get $s_{5}\left(\tau ,\eta \right)$ by substituting the solution of SP into the IFFT form of $s_{4}\left(\tau ,\eta \right)$:

(23) $$\begin{array}{cc} s_{5}\left(\tau ,\eta \right) & =\int_{f_{\eta }=-\infty }^{+\infty }s_{4}\left(\tau ,f_{\eta }\right)\exp\left(j2\pi f_{\eta }\eta \right)df_{\eta }\\ & \approx {s_{4}\left(\tau ,f_{\eta }\right)\exp\left(j2\pi f_{\eta }\eta \right)|}_{f_{\eta }=K_{a}\left(\eta -\eta_{p}\right)+\frac{3}{2}k_{3}K_{a}^{3}{\left(\eta -\eta_{p}\right)}^{2}}\\ & =\omega_{a}\left(\eta -\eta_{c}\right)\mathrm{sinc}\left(\tau -2R_{LRWC}/c\right)\exp\left(j\pi \theta_{5}\left(f_{\eta }\right)\right) \end{array}$$

$\theta_{5}\left(\eta \right)$ is rewritten as:

(24) $$\begin{array}{cc} \theta_{5}\left(\eta \right) & =\pi K_{a}{\left(\eta -\eta_{p}\right)}^{2}+\pi k_{3}K_{a}^{3}{\left(\eta -\eta_{p}\right)}^{3}+\frac{1}{4}\pi K_{a}^{4}\left(4k_{4}+9k_{3}^{2}K_{a}\right){\left(\eta -\eta_{p}\right)}^{4}\\ & +\frac{1}{4}\pi K_{a}^{5}\left(4k_{5}+3k_{3}K_{a}\left(8k_{4}+9k_{3}^{2}K_{a}\right)\right){\left(\eta -\eta_{p}\right)}^{5}\\ & =\pi K_{a}{\left(\eta -\eta_{p}\right)}^{2}+\pi \sum_{i=3}^{5}p_{i}{\left(\eta -\eta_{p}\right)}^{i} \end{array}$$

The coefficients are as follows:

(25) $$\begin{array}{l} k_{i}=Y_{i}+\varphi_{i,s}^{\prime }\eta_{p}\left(i=3,4,5\right)\\ p_{3}=k_{3}K_{a}^{3}\\ p_{4}=\frac{1}{4}K_{a}^{4}\left(4k_{4}+9k_{3}^{2}K_{a}\right)\\ p_{5}=\frac{1}{4}K_{a}^{5}\left(4k_{5}+3k_{3}K_{a}\left(8k_{4}+9k_{3}^{2}K_{a}\right)\right) \end{array}$$

To compensate the azimuth variance and the difference of Doppler rates along the azimuth, a high-order nonlinear azimuth chirp scaling filter is applied:

(26) $$H_{4}\left(\eta \right)=\exp\left(j\pi \sum_{i=2}^{5}q_{i}\eta^{i}\right)$$

Multiplying (26) with (23) results in $s_{6}\left(\tau ,\eta \right)$:

(27) $$\begin{array}{cc} s_{6}\left(\tau ,\eta \right) & =s_{5}\left(\tau ,\eta \right)H_{4}\left(\eta \right)\\ & =\omega_{a}\left(\eta -\eta_{c}\right)\mathrm{sinc}\left(\tau -2R_{LRWC}/c\right)\exp\left(j\pi \theta_{5}\left(f_{\eta }\right)+j\pi \sum_{i=2}^{5}q_{i}\eta^{i}\right) \end{array}$$

In order to achieve a fast calculation for azimuth compression, an FFT for the Equation (27) yields the $s_{7}\left(\tau ,f_{\eta }\right)$ in RD domain. Also, the solution of SP is calculated by MSR:

(28) $$\begin{array}{cc} \eta_{SP} & =\frac{f_{\eta }+\eta_{p}K_{a}+\sum_{i=3}^{5}\frac{i}{2}{\left(-\eta_{p}\right)}^{i-1}p_{i}}{K_{a}+\sum_{i=3}^{5}i\left(i-1\right){\left(-\eta_{p}\right)}^{i-1}p_{i}+q_{2}}\\ & -\frac{\left(\sum_{i=3}^{5}P_{i}^{3}p_{i}{\left(-\eta_{p}\right)}^{i-3}+6q_{3}\right){\left(f_{\eta }+\eta_{p}K_{a}+\sum_{i=3}^{5}\frac{i}{2}{\left(-\eta_{p}\right)}^{i-1}p_{i}\right)}^{2}}{4{\left(K_{a}+\sum_{i=3}^{5}i\left(i-1\right){\left(-\eta_{p}\right)}^{i-1}p_{i}+q_{2}\right)}^{3}} \end{array}$$

Expanding (28) near $\eta_{p}=0$ by the principle of Taylor expansion yields the results:

(29) $$\left\{ \begin{array}{l} \frac{1}{K_{a}+\sum_{i=3}^{5}i\left(i-1\right){\left(-\eta_{p}\right)}^{i-1}p_{i}+q_{2}}\approx \frac{1}{K_{LRWC}+q_{2}}+\frac{\left(-K_{LRWC}K_{s}+3K_{LRWC}^{3}X_{3}\right)\eta_{p}}{{\left(K_{LRWC}+q_{2}\right)}^{2}}\\ +\frac{\left(-2K_{LRWC}K_{s}^{2}q_{2}+6K_{LRWC}^{4}K_{s}X_{3}+18K_{LRWC}^{3}K_{s}q_{2}X_{3}-9K_{LRWC}^{6}X_{3}^{2}\right)\eta_{p}^{2}}{2{\left(K_{LRWC}+q_{2}\right)}^{3}}\\ \frac{1}{{\left(K_{a}+\sum_{i=3}^{5}i\left(i-1\right){\left(-\eta_{p}\right)}^{i-1}p_{i}+q_{2}\right)}^{3}}\approx \frac{1}{{\left(K_{LRWC}+q_{2}\right)}^{3}}-\frac{3\left(K_{LRWC}K_{s}-3K_{LRWC}^{3}X_{3}\right)\eta_{p}}{{\left(K_{LRWC}+q_{2}\right)}^{4}}\\ +\frac{\frac{12{\left(K_{LRWC}K_{s}-3K_{LRWC}^{3}X_{3}\right)}^{2}}{{\left(K_{LRWC}+q_{2}\right)}^{2}}-\frac{6\left(K_{LRWC}K_{s}^{2}-9K_{LRWC}^{3}K_{s}X_{3}+\frac{27}{2}K_{LRWC}^{5}Y_{3}^{2}+6K_{LRWC}^{4}X_{4}\right)}{K_{LRWC}+q_{2}}\eta_{p}^{2}}{2{\left(K_{LRWC}+q_{2}\right)}^{3}} \end{array}\right.$$

Then we can get the phase $\theta_{7}\left(f_{\eta }\right)$ of the $s_{7}\left(\tau ,f_{\eta }\right)$ after the Taylor expansion:

(30) $$\begin{array}{cc} \theta_{7}\left(f_{\eta }\right) & =A\left(q_{2},q_{3},q_{4},q_{5},X_{3},X_{4},X_{5},f_{\eta },f_{\eta }^{2},f_{\eta }^{3},f_{\eta }^{4},f_{\eta }^{5}\right)\\ & +B\left(q_{2},q_{3},q_{4},q_{5},X_{3},X_{4},X_{5}\right)\eta_{p}f_{\eta }+C\left(q_{2},q_{3},q_{4},q_{5},X_{3},X_{4},X_{5}\right)\eta_{p}^{2}f_{\eta }\\ & +D\left(q_{2},q_{3},q_{4},q_{5},X_{3},X_{4},X_{5}\right)\eta_{p}f_{\eta }^{2}+E\left(q_{2},q_{3},q_{4},q_{5},X_{3},X_{4},X_{5}\right)\eta_{p}^{2}f_{\eta }^{2}\\ & +F\left(q_{2},q_{3},q_{4},q_{5},X_{3},X_{4},X_{5}\right)\eta_{p}f_{\eta }^{3}+G\left(q_{2},q_{3},q_{4},q_{5},X_{3},X_{4},X_{5}\right)\eta_{p}^{2}f_{\eta }^{3}\\ & +H\left(q_{2},q_{3},q_{4},q_{5},X_{3},X_{4},X_{5}\right)\eta_{p}f_{\eta }^{4}+\phi_{res}\left(q_{2},q_{3},q_{4},q_{5},X_{3},X_{4},X_{5}\right) \end{array}$$

The first term on the right of (30) is the azimuth frequency modulation. The second term is the real target azimuth position. The third–the eighth term represents the nonlinear geometric deviation along the azimuth direction. The last term, $\phi_{res}$, is the residual phase, which needs to be eliminated in the applications, e.g., SAR interferometry and differential SAR interferometry. The coefficients are shown in Table 1.

The coefficients of $q_{i}\left(i=2,3,4,5\right)$ and $X_{i}\left(i=3,4,5\right)$ can be solved by the following equations:

(31) $$\left\{ \begin{array}{l} B\left(q_{2},q_{3},q_{4},q_{5},X_{3},X_{4},X_{5}\right)=-\frac{\pi }{\alpha }\\ C\left(q_{2},q_{3},q_{4},q_{5},X_{3},X_{4},X_{5}\right)=0\\ D\left(q_{2},q_{3},q_{4},q_{5},X_{3},X_{4},X_{5}\right)=0\\ E\left(q_{2},q_{3},q_{4},q_{5},X_{3},X_{4},X_{5}\right)=0\\ F\left(q_{2},q_{3},q_{4},q_{5},X_{3},X_{4},X_{5}\right)=0\\ G\left(q_{2},q_{3},q_{4},q_{5},X_{3},X_{4},X_{5}\right)=0\\ H\left(q_{2},q_{3},q_{4},q_{5},X_{3},X_{4},X_{5}\right)=0 \end{array}\right.$$

The parameters are solved by Equation (31):

(32) $$\left\{ \begin{array}{l} q_{2}=(-1+2\alpha )K_{LRWC}\\ q_{3}=\frac{1}{3}(-1+2\alpha )K_{LRWC}K_{s}\\ q_{4}=\frac{K_{LRWC}}{12}\left(K_{s}^{2}\left(-3+7\alpha \right)-3K_{LRWC}^{2}\varphi_{3,s}^{\prime }\left(1-2\alpha \right)\right)\\ q_{5}=\frac{K_{LRWC}}{60(-1+2\alpha )}\left((9-50\alpha +82\alpha^{2})K_{s}^{3}+6K_{LRWC}^{2}K_{s}\varphi_{3,s}^{\prime }(5-24\alpha +25\alpha^{2})+12K_{LRWC}^{3}\varphi_{4,s}^{\prime }{\left(1-2\alpha \right)}^{2}\right)\\ X_{3}=\frac{(-1+4\alpha )K_{s}}{3(-1+2\alpha )K_{LRWC}^{2}}\\ X_{4}=\frac{-6\alpha K_{s}^{2}-3K_{LRWC}^{2}\varphi_{3,s}^{\prime }\left(1-4\alpha \right)}{12(-1+2\alpha )K_{LRWC}^{3}}\\ X_{5}=\frac{-3\alpha K_{s}\varphi_{3,s}^{\prime }-K_{LRWC}\varphi_{4,s}^{\prime }\left(1-4\alpha \right)}{5(-1+2\alpha )K_{LRWC}^{2}} \end{array}\right.$$

The coefficient $A\left(q_{2},q_{3},q_{4},q_{5},X_{3},X_{4},X_{5},f_{\eta },f_{\eta }^{2},f_{\eta }^{3},f_{\eta }^{4},f_{\eta }^{5}\right)$ is rewritten as:

(33) $$A\left(q_{2},q_{3},q_{4},q_{5},X_{3},X_{4},X_{5},f_{\eta },f_{\eta }^{2},f_{\eta }^{3},f_{\eta }^{4},f_{\eta }^{5}\right)=\sum_{i=1}^{5}A_{i}\left(q_{2},q_{3},q_{4},q_{5},X_{3},X_{4},X_{5}\right)f_{\eta }^{i}$$

Table 2 shows the expressions of the coefficients $A_{i}\left(i=1\sim 5\right)$.

The azimuth compression filter is given as follow:

(34) $$H_{5}\left(f_{\eta }\right)=\exp\left(-\sum_{i=2}^{5}A_{i}\left(q_{2},q_{3},q_{4},q_{5},X_{3},X_{4},X_{5}\right)f_{\eta }^{i}\right)$$

The principle of stationary phase is applied to derive the signal after azimuth compression in the RD domain. The signal in the azimuth time domain is expressed as:

(35) $$s\left(\tau ,\eta \right)=\mathrm{sinc}\left(\tau -\frac{2R_{LRWC}}{c}\right)\mathrm{sinc}\left(\eta -\frac{\eta_{p}}{2\alpha }\right)\exp\left(-j2\pi \eta_{p}\eta \right)$$

From the Equation (35), it obtains that the target position after focusing is shifted to $\left(R_{0}+x_{p}\sin\theta_{s},x_{p}/\left(2\alpha \right)\right)$. Readers can adopt the method of geometric correction to correct the geometric shift, which has been illustrated in [1].

The generalized chirp scaling algorithm in [4] is the special form of GNLCSA in the case of $\theta_{s}=0$. The difference between ENLCSA and GNLCSA is that the coefficients and the phase of azimuth compression in GNLCSA framework are more precise than ones of ENLCSA.

### 2.3. Block Diagram of GNLCSA

**Figure 3:**
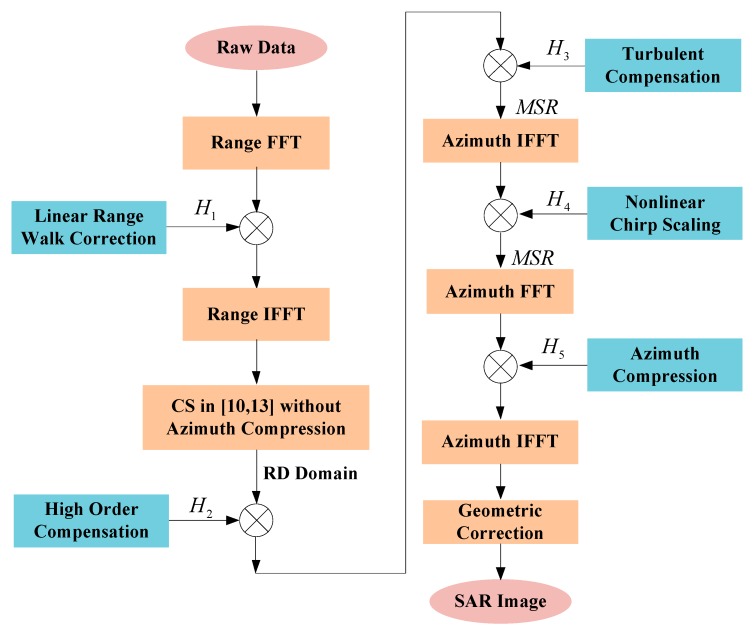
Block diagram of proposed generalized nonlinear chirp scaling (GNLCSA).

The blocks diagram of the proposed GNLCSA is shown in the Figure 3.

The steps of the proposed algorithm for high-resolution highly squint SAR are as follows:

Step 1. A range FFT is used to transfer the raw data to the range frequency domain. The data is multiplied with the filter of LRWC to achieve the processing of “equivalent broadside” in range direction.
Step 2. range IFFT is implemented to transfer the data after LRWC into the range time domain. The modified CSA proposed in [18,21], is introduced to process the former high-resolution squint data, which has the advantages of eliminating the coupling between the range and azimuth dimensions and enhancing the focusing precision for the range dimension at any ratio of $B_{r}/f_{0}$ ($B_{r}$ is the bandwidth of the transmitting pulse).
Step 3. The high order compensation filter is multiplied with the data after the processing of step 2. It reduces the deteriorations of focusing caused by the high order phase error.
Step 4. A turbulent compensation filter is multiplied with the data after the processing of step 3 to achieve a coincident FM rate form of different slant range along the azimuth direction.
Step 5. The MSR is implemented in the azimuth IFFT for the data after the processing of step 4. Then a nonlinear chirp scaling filter is applied to compensate the azimuth variance.
Step 6. After the processing of the nonlinear chirp scaling, the MSR is also applied in the azimuth FFT for the data. The azimuth compression filter is used to achieve the focusing of the squint data. Then an IFFT is applied for the focusing data in the RD domain.
Step 7. The geometric correction is applied in the final step to obtain images matching the geometry of the signal acquisition.

## 3. Experimental Results and Discussion

### 3.1. Experimental Results

The simulation is carried out with the parameters of Table 3.

**Figure 4:**
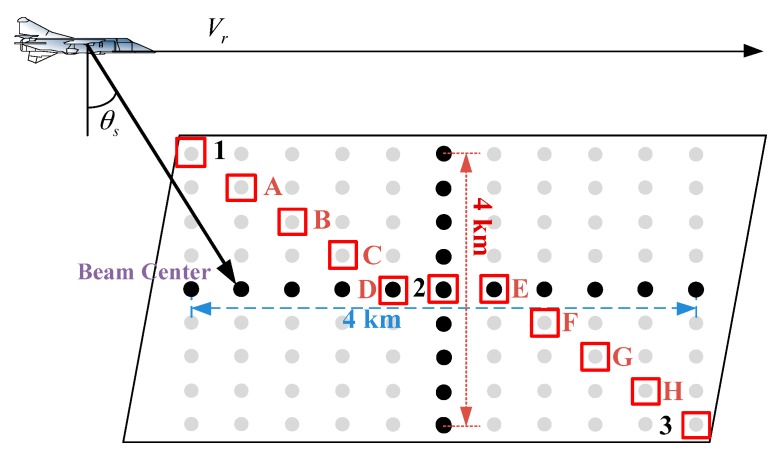
Flight geometry and dot-matrix targets.

The flight geometry and dot-matrix targets are shown in the Figure 4.

**Figure 5:**
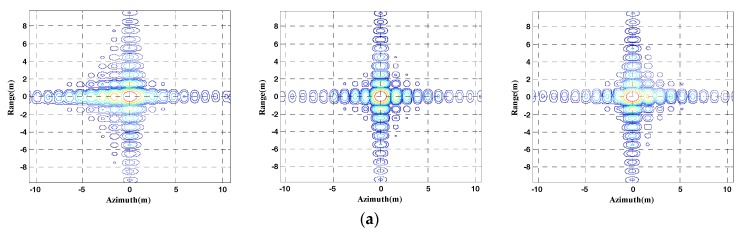

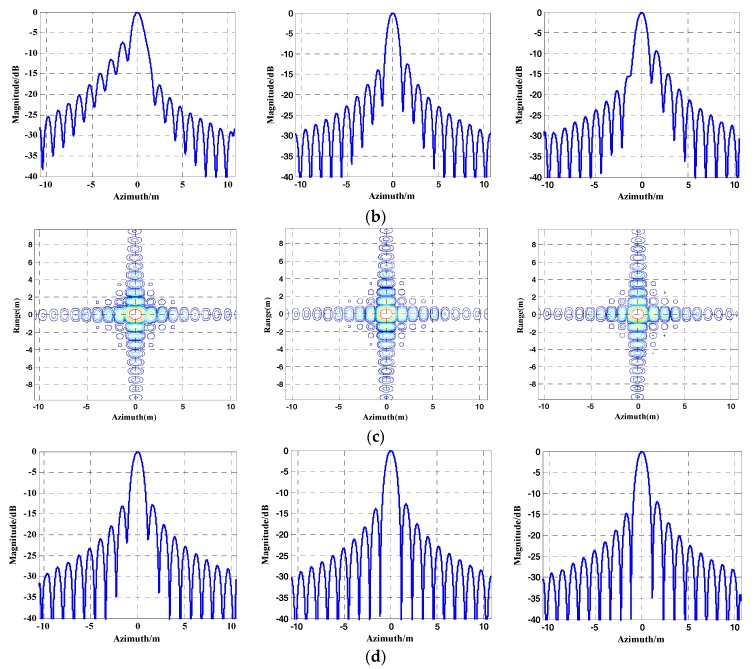
Simulation results processed by different algorithms. The sub-images in each column correspond to target 1, target 2, and target 3, respectively. (**a**,**c**) and (**b**,**d**) show the contours azimuth slice of the results processed by the extended nonlinear CS algorithm (ENLCSA) and generalized nonlinear chirp scaling (GNLCSA), respectively.

The gray dots in Figure 4 represent the dot-matrix targets. To make a clear and fair comparison, no weighting functions or methods of side-lobe controlling is applied in the algorithms. The results processed by different algorithms are shown in Figure 5.

The three targets focusing performances processed by different algorithms are shown in Table 4, where the focusing performances include resolution (Res), peak side-lobe ratio (PSLR), and integrated side-lobe ratio (ISLR).

When comparing the results and performances in Figure 5 and Table 4, it is obvious that the results achieved by GNLCSA are better than the ones that are processed by ENLCSA. In Figure 5, the sub-images shown in (a) and (b) indicate that azimuth FM rates are mismatched during the processing of ENLCSA. The quadratic and cubic phases still exist in a large proportion, which causes the deterioration of azimuth compression. The focusing performance tends to be worse when the distance bias that is away from the referenced azimuth position becomes larger. The sub-images listed in Figure 5c,d are well focused. It is concluded that the azimuth variant FM rates and high orders are eliminated effectively in different azimuth position, and the azimuth focusing quality and the ADOF are greatly improved by GNLCSA.

**Figure 6:**
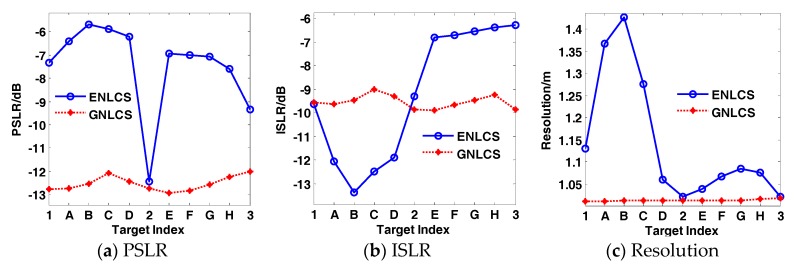
Focusing performances of different algorithms on 11 points marked in Figure 4. The blue solid lines with ‘o’ marker are the results of ENLCSA. The red dash lines with ‘*’ marker are the results of the GNLCSA. (**a**–**c**) represent the peak side-lobe ratio (PSLR), integrated side-lobe ratio (ISLR) and resolution, respectively.

We selected eight points with different azimuth and range position in Figure 4, and signed with A, B, C, D, E, F, G, and H, respectively. Comparison of the focusing performances of GNLCSA and ENLCSA on points 1–3 and A–H is performed. The results are shown in Figure 6.

The focusing performances of the 11 points marked in Figure 4 are shown in Figure 6.

The normalized values of PSLR and ISLR for a precise focusing point (the antenna pattern is a rectangle) are −13.27 dB and −9.60 dB, respectively. From Figure 6, it can be concluded that the focusing performances of the GNLCSA are much better than the ones of the ENLCSA. PSLR and ISLR of GNLCSA are close to the normalized value. In addition, the GNLCSA is also more stable than the ENLCSA.

Video SAR has great advantages in the applications of ground moving target indication (GMTI), for it can form a series of consecutive frames. Video SAR needs images at different squint angle to form the video sequence. Also, it requires a high focusing quality and a fast imaging ability for each frame. The simulation results show that the ENLCSA is not accessible to the focusing requirements of the scene size (4 km × 4 km) for squint SAR (the squint angle is 45 degree). It means that the ENLCSA cannot be applied in the video SAR with the parameters in Table 3, but the GNLCSA can be done.

### 3.2. Discussion

As the azimuth variance of equivalent range cannot be formatted in the RD domain, the approximation is adopted during the compensation of high order phase in the filter of $H_{2}\left(f_{\eta }\right)$. The phase error of the compensation plays an important role in the AODF and the focusing precision, which can be expressed as:

(36) $$\Delta \Psi_{error}=\left(\sum_{i=3}^{\infty }\varphi_{i,LRWC}\left(R_{LRWC}\right)-\sum_{i=3}^{\infty }\varphi_{i,LRWC}\left(R_{0}\right)\right)f_{\eta }^{i}$$

**Figure 7:**
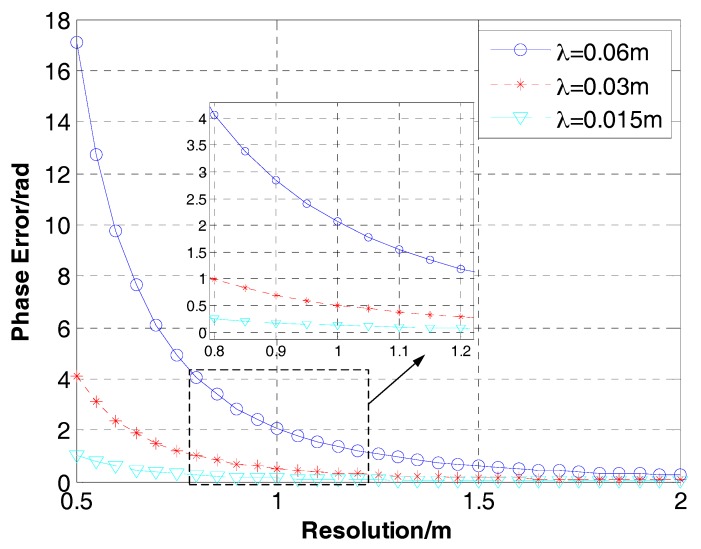
Phase error of targets in the edge (the distance departing from the referenced azimuth position is 2 km and the squint angle is 45°) varies with the azimuth resolution and wavelength. The blue solid line with ‘o’, the red dash line with ‘*’ and the cyan lineation line with ‘∇’ are the results with the wavelength equaling to 0.06 m, 0.03 m and 0.015 m, respectively.

**Figure 8:**
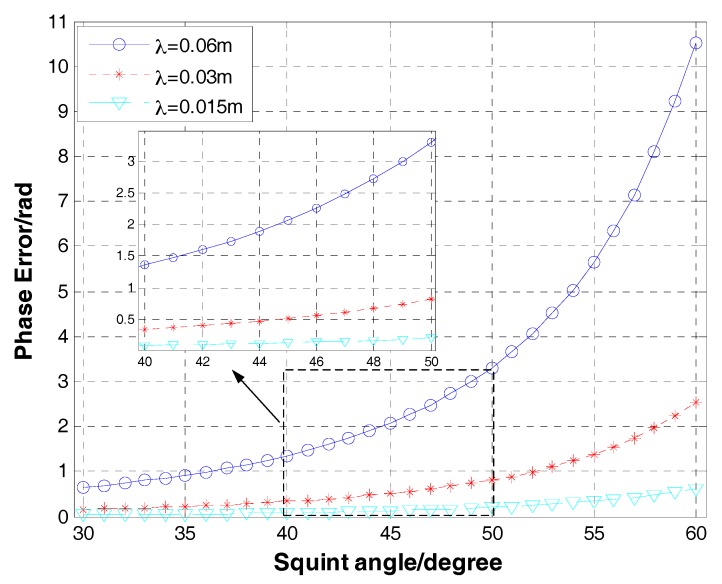
Phase error of targets in the edge (the distance departing from the referenced azimuth position is 2 km and the azimuth resolution is 1 m) varies with the squint angle and wave length. The blue solid line with ‘o’, the red dash line with ‘*’ and the cyan lineation line with ‘∇’ are the results of the wavelength equaling to 0.06 m, 0.03 m and 0.015 m, respectively.

According to the Equation (36), we do the simulations to inquire the potential limitations of GNLCSA. Figure 7 and Figure 8 show the phase error of targets in the edge (the distance depart from the referenced azimuth position is 2 km), varying with the azimuth resolution or the squint angle at different wavelength.

From Figure 7 and Figure 8, it is obvious that the phase error decreases when the wavelength descends. In the process of SAR imaging, it can be neglected if the phase error is smaller than π/4. In general, it can conclude that the GNLCSA is invalid in the occasions that the wavelength is greater than 0.06 m. Furthermore, the GNLCSA is unavailable for the applications of the low carrier frequency. It will greatly decrease the AODF and the focusing precision in those occasions. Contrastively, the GNLCSA can be applied in the occasions (the squint angle can be up to 60° and the azimuth resolution can be smaller than 0.8 m) when the wavelength is smaller than 0.015 m.

## 4. Conclusions

With the advantages of flexible observation, the squint SAR with the multi-mode is widely used to accomplish different monitoring missions. The GNLCSA proposed in this paper derived the new perturbation function and the nonlinear chirp scaling function by the method of series reversion. Also, it modifies the procedures of the processing in the range dimension to avoid the tedious range block processing in ENLCSA [1]. In general, from the perspective of the algorithmic framework, the NLCSA and ENLCSA are special forms of the proposed algorithm. The simulation results indicate that the ADOF and the focusing precise have been well improved in the areas apart from the azimuth reference position with the process of the GNLCSA.

## Figures and Tables

**Table 1:** Coefficients for different order.

Coefficients	Value
$B\left(q_{2},q_{3},q_{4},q_{5},X_{3},X_{4},X_{5}\right)$	$-\frac{2\pi K_{LRWC}}{K_{LRWC}+q_{2}}$
$C\left(q_{2},q_{3},q_{4},q_{5},X_{3},X_{4},X_{5}\right)$	$\frac{\pi K_{LRWC}\left(K_{LRWC}K_{s}+K_{s}q_{2}+3q_{3}-3K_{1}^{2}q_{2}X_{3}\right)}{{\left(K_{LRWC}+q_{2}\right)}^{3}}$
$D\left(q_{2},q_{3},q_{4},q_{5},X_{3},X_{4},X_{5}\right)$	$\pi K_{LRWC}\frac{-2K_{s}q_{2}^{2}+K_{LRWC}(-2K_{s}q_{2}+3q_{3})+3K_{LRWC}^{2}q_{2}^{2}X_{3}}{{\left(K_{LRWC}+q_{2}\right)}^{3}}$
$E\left(q_{2},q_{3},q_{4},q_{5},X_{3},X_{4},X_{5}\right)$	$\frac{\pi K_{LRWC}}{2{\left(K_{LRWC}+q_{2}\right)}^{5}}\left(\begin{array}{l} 2K_{s}q_{2}^{2}(K_{s}q_{2}+3q_{3})+K_{LRWC}\left(4K_{s}^{2}q_{2}^{2}-6K_{s}q_{2}q_{3}-27q_{3}^{2}+12q_{2}q_{4}\right)\\ -3K_{LRWC}^{4}\left(3q_{3}X_{3}+q_{2}(-9q_{2}^{2}X_{3}^{2}-4q_{2}X_{4}+2\psi_{3,s}^{\prime })\right) \end{array}\right)$
$F\left(q_{2},q_{3},q_{4},q_{5},X_{3},X_{4},X_{5}\right)$	$\frac{-\pi K_{LRWC}}{{\left(K_{LRWC}+q_{2}\right)}^{5}}\left(\begin{array}{l} 3K_{s}q_{2}q_{3}+9q_{3}^{2}+K_{LRWC}(3K_{s}q_{3}-4q_{4})+K_{LRWC}^{4}(9q_{2}^{2}X_{3}^{2}+4q_{2}X_{4}-\psi_{3,s}^{\prime })\\ -K_{LRWC}^{2}q_{2}(3K_{s}q_{2}X_{3}+9q_{3}X_{3}+q_{2}\psi_{3,s}^{\prime }) \end{array}\right)$
$G\left(q_{2},q_{3},q_{4},q_{5},X_{3},X_{4},X_{5}\right)$	$\frac{\pi K_{LRWC}}{2{\left(K_{LRWC}+q_{2}\right)}^{7}}\left(\begin{array}{l} -6K_{LRWC}K_{s}^{2}q_{2}^{2}q_{3}+K_{s}\left(54q_{2}q_{3}^{2}-8q_{2}^{2}q_{4})+5(27q_{3}^{3}-24q_{2}q_{3}q_{4}+4q_{2}^{2}q_{5})\right)\\ +K_{LRWC}^{3}\left(40q_{2}q_{5}+27q_{2}^{2}q_{3}^{2}X_{3}+6K_{s}^{2}q_{2}(q_{3}+2q_{2}^{3}X_{3})\right)\\ +2K_{LRWC}^{4}\left(10q_{5}-K_{s}q_{2}^{2}(27q_{3}X_{3}+16q_{2}^{2}X_{4}-9q_{2}\varphi_{3,s}^{\prime })\right)\\ +q_{2}\left(30q_{2}q_{4}X_{3}+9q_{2}q_{3}(-2q_{2}X_{4}+\varphi_{3,s}^{\prime })-4q_{2}^{3}\varphi_{4,s}^{\prime })\right)\\ -4K_{LRWC}^{5}\left(-9q_{2}q_{4}X_{3}+q_{2}^{4}(-5X_{5}+9X_{3}\varphi_{3,s}^{\prime })+6q_{2}^{3}\varphi_{4,s}^{\prime }\right) \end{array}\right)$
$H\left(q_{2},q_{3},q_{4},q_{5},X_{3},X_{4},X_{5}\right)$	$\frac{\pi K_{LRWC}}{4{\left(K_{LRWC}+q_{2}\right)}^{7}}\left(\begin{array}{l} K_{s}q_{2}\left(45q_{3}^{2}-16q_{2}q_{4}\right)+K_{LRWC}\left(54q_{2}q_{3}^{2}-8q_{2}^{2}q_{4})+5(27q_{3}^{3}-24q_{2}q_{3}q_{4}+4q_{2}^{2}q_{5})\right)\\ +5\left(27q_{3}^{3}-24q_{2}q_{3}q_{4}+4q_{2}^{2}q_{5}\right)+K_{LRWC}^{5}\left(K_{s}q_{2}\left(45q_{2}X_{3}^{2}+16X_{4}\right)-48q_{3}X_{4}-120q_{2}^{3}X_{3}X_{4}\right)\\ +K_{LRWC}^{2}\left(27q_{3}X_{3}^{2}+q_{2}\left(-20X_{5}+18X_{3}\varphi_{3,s}^{\prime }\right)+4\varphi_{4,s}^{\prime }\right)+K_{LRWC}^{4}K_{s}\left(36q_{3}X_{3}+45q_{2}^{3}X_{3}^{2}+32q_{2}^{2}X_{4}\right)\\ +2K_{LRWC}^{4}K_{s}\left(q_{2}^{3}(-10X_{5}+9X_{3}\varphi_{3,s}^{\prime })+q_{2}^{2}(81q_{3}X_{3}^{2}+6\varphi_{4,s}^{\prime })\right) \end{array}\right)$

**Table 2:** Coefficients values in Equation (33).

Coefficients	Value
$A_{1}\left(q_{2},q_{3},q_{4},q_{5},X_{3},X_{4},X_{5}\right)$	0
$A_{2}\left(q_{2},q_{3},q_{4},q_{5},X_{3},X_{4},X_{5}\right)$	$-\frac{\pi }{K_{LRWC}+q_{2}}$
$A_{3}\left(q_{2},q_{3},q_{4},q_{5},X_{3},X_{4},X_{5}\right)$	$\frac{\pi (q_{3}+K_{LRWC}^{3}X_{3})}{{\left(K_{LRWC}+q_{2}\right)}^{3}}$
$A_{4}\left(q_{2},q_{3},q_{4},q_{5},X_{3},X_{4},X_{5}\right)$	$\frac{\pi }{4{\left(K_{LRWC}+q_{2}\right)}^{5}}\left(4(K_{LRWC}+q_{2})(q_{4}+K_{LRWC}^{4}X_{4})-9(q_{3}^{2}+2K_{LRWC}^{3}q_{3}X_{3}-K_{LRWC}^{5}q_{2}X_{3}^{2})\right)$
$A_{5}\left(q_{2},q_{3},q_{4},q_{5},X_{3},X_{4},X_{5}\right)$	$\frac{\pi }{4{\left(K_{LRWC}+q_{2}\right)}^{7}}\left(\begin{array}{l} 4{\left(K_{LRWC}+q_{2}\right)}^{2}(q_{5}+K_{LRWC}^{5}X_{5})+27q_{3}{\left(q_{3}+K_{LRWC}^{3}X_{3}\right)}^{2}\\ +24(K_{LRWC}+q_{2})\left(\begin{array}{l} -q_{3}(q_{4}+K_{LRWC}^{4}X_{4})\\ +K_{LRWC}^{3}X_{3}(-q_{4}+K_{LRWC}^{3}q_{2}X_{4}) \end{array}\right)+27K_{LRWC}^{3}X_{3}{\left(q_{3}-K_{LRWC}^{2}q_{2}X_{3}\right)}^{2} \end{array}\right)$

**Table 3:** Parameters of simulation.

Parameters	Values
Center Frequency ($f_{0}$)	8 GHz
Bandwidth ($B_{r}$)	150 MHz
Sampling Frequency ($f_{s}$)	180 MHz
Antenna Size ($D_{r}\times D_{a}$)	2 m × 1.5 m
Scene Size/km	4 km × 4 km
Targets Distribution (R × A)	9 × 11
Sensor Velocity ($V_{r}$)	100 m/s
Pulse Repeat Frequency (PRF)	300 Hz
Pulse Width ($T_{r}$)	30 us
Center Slant Range ($R_{0}$)	20 km
Squint Angle ($\theta_{s}$)	45°

**Table 4:** Focusing performances for ENLCSA or GNLCSA.

Algorithm	Dimension	Performance	Target Index		
			1	2	3
ENLCSA	Range	Res/m	0.8854	0.8854	0.8854
		PSLR/dB	−13.2545	−13.2675	−13.2818
		ISLR/dB	−9.9274	−9.8464	−9.8847
	Azimuth	Res/m	1.1294	1.0221	1.0219
		PSLR/dB	−7.3320	−12.4446	−9.3459
		ISLR/dB	−9.6334	−9.3022	−6.2756
GNLCSA	Range	Res/m	0.8828	0.8854	0.8854
		PSLR/dB	−13.2864	−13.2761	−13.3001
		ISLR/dB	−9.3836	−9.8400	−9.8404
	Azimuth	Res/m	1.0115	1.0135	1.0188
		PSLR/dB	−12.7645	−12.7299	−12.0016
		ISLR/dB	−9.5608	−9.6637	−9.0291

## References

[1] An D., Huang X., Jin T., Zhou Z. (2012). Extended Nonlinear Chirp Scaling Algorithm for High-Resolution Highly Squint SAR Data Focusing. IEEE Trans. Geosci. Remote Sens..

[2] Carrara W.G., Goodman R.S., Majewski R.M. (1997). Signal properties of spaceborne squint-mode SAR. IEEE Trans. Geosci. Remote Sens..

[3] Wong F.H., Cumming I.G., Neo Y.L. (2008). Focusing bistatic SAR datausing the nonlinear chirp scaling algorithm. IEEE Trans. Geosci. Remote Sens..

[4] Jin M.Y., Wu C. (1984). A SAR correlation algorithm which accommodates large range migration. IEEE Trans. Geosci. Remote Sens..

[5] Bamler R. (1992). A comparison of range-Doppler and wavenumber domain SAR focusing algorithms. IEEE Trans. Geosci. Remote Sens..

[6] Wang K., Liu X. (2007). Quartic-phase algorithm for highly squinted SAR data processing. IEEE Geosci. Remote Sens. Lett..

[7] Yamaoka T., Suwa K., Hara T., Nakano Y. Radar Video Generated from Synthetic Aperture Radar Image. Proceedings of the International Geoscience Remote Sensing Symposium.

[8] Bishop E., Linnehan R. Video-SAR Using Higher Order Taylor Terms for Differential Range. Proceedings of the IEEE Radar Conference.

[9] Liu B., Zhang X., Tang K., Liu M., Liu L. Spaceborne Video-SAR Moving Target Surveillance System. Proceedings of the International Geoscience Remote Sensing Symposium.

[10] Song X., Yu W. (2016). Derivation and application of stripmap VideoSAR parameter relations. J. Univ. Chin. Acad. Sci..

[11] Li C., He M. (2017). A Generalized Chirp-Scaling Algorithm for Geosynchronous Orbit SAR Staring Observations. Sensors.

[12] Hua L. (2010). Analysis and simulation of UAV terahertz wave synthetic aperture radar imaging. Inf. Electron. Eng..

[13] Moreira A., Huang Y. (1994). Airbome SAR Processing of Highly Squinted Data Using a Chirp Scaling Approach with Integrated Motion Compensation. IEEE Trans. Geosci. Remote Sens..

[14] Raney R.K., Runge H., Bamler R., Cumming I.G., Wong F.H. (1994). Precision SAR processing using chirp scaling. IEEE Trans. Geosci. Remote Sens..

[15] Firooz S. New comparative experiments in range migration mitigation methods using polarimetric inverse synthetic aperture radar signatures of small boats. Proceedings of the Radar Conference.

[16] Cafforio C., Prati C., Rocca F. (1991). SAR data focusing using seismic migration techniques. IEEE Trans. Aerosp. Electron. Syst..

[17] Li D., Lin H., Liu H., Liao G., Tan X. (2017). Focus Improvement for High-Resolution Highly Squinted SAR Imaging Based on 2-D Spatial-Variant Linear and Quadratic RCMs Correction and Azimuth-Dependent Doppler Equalization. IEEE J. Sel. Top. Appl. Earth Obs. Remote Sens..

[18] Yi T., He Z., He F., Dong Z., Wu M. (2017). Generalized Chirp Scaling Combined with Baseband Azimuth Scaling Algorithm for Large Bandwidth Sliding Spotlight SAR Imaging. Sensors.

[19] An D., Huang X., Zhou Z. Extended Wavenumber Domain Algorithm for Airborne Low Frequency SAR in Highly Squinted Mode. Proceedings of the IET International Radar Conference.

[20] Wong F.H., Yeo T.S. (2001). New Application of Nonlinear Chirp Scaling in SAR Data Processing. IEEE Trans. Geosci. Remote Sens..

[21] Zaugg E.C., Long D.G. (2009). Generalized Frequency-Domain SAR Processing. IEEE Trans. Geosci. Remote Sens..

